# Factors associated with dengue preventive attitudes and practices: a cross-sectional study in Peru

**DOI:** 10.15649/cuidarte.4626

**Published:** 2026-05-15

**Authors:** Irma Luz Yupari-Azabache, Nélida Milly Otiniano, Lucia Beatriz Bardales-Aguirre, Susana Edita Paredes-Díaz

**Affiliations:** 1 Universidad César Vallejo. Institutes and Research Centers, Trujillo, Peru. E-mail: iyupari@ucv.edu.pe Universidad César Vallejo Trujillo Peru iyupari@ucv.edu.pe; 2 Universidad César Vallejo. Institutes and Research Centers, Trujillo, Peru. E-mail: notiniano@ucv.edu.pe Universidad César Vallejo Trujillo Peru notiniano@ucv.edu.pe; 3 Universidad Privada del Norte, Trujillo, Peru. E-mail: lucia.bardales@upn.edu.pe Universidad Privada del Norte Trujillo Peru lucia.bardales@upn.edu.pe; 4 Universidad César Vallejo. Institutes and Research Centers, Trujillo, Peru. E-mail: sparedes@ucv.edu.pe Universidad César Vallejo Trujillo Peru sparedes@ucv.edu.pe

**Keywords:** Dengue, Primary Prevention, Attitudes, Virus, Aedes, Dengue, Prevención Primaria, Actitudes, Virus, Aedes, Dengue, Prevenção Primária, Atitudes, Vírus, Aedes

## Abstract

**Introduction:**

Dengue is a viral disease transmitted through the bite of infected mosquitoes, primarily Aedes aegypti, which may be fatal in severe cases. Studying the population's preventive attitudes and practices is essential for strengthening control and prevention strategies.

**Objective:**

To analyze the factors associated with dengue preventive attitudes and practices among citizens of northern Peru.

**Materials and Methods:**

A quantitative, non-experimental, cross-sectional, descriptive, and correlational study was conducted. A total of 295 citizens aged 18 years and older participated, selected through non-probability sampling. A validated 67-item questionnaire was used, with a dichotomous scale for knowledge and a Likert-type scale for attitudes and practices. Descriptive and inferential analyses (Cramer's V and Kendall's Tau-b) were performed with a 95% confidence level.

**Results:**

A total of 49.5% showed indifference toward dengue prevention, 93.6% demonstrated adequate practices, and 54.6% exhibited a high level of overall knowledge about dengue prevention.

**Discussion:**

The most commonly identified attitude was fear that a family member might contract the disease, while the most frequent practices included eliminating mosquito breeding sites by changing the water in plant saucers and using commercial insect repellents. These findings are similar to those reported in previous studies.

**Conclusion:**

A significant association was found between preventive attitudes and practices and most cultural dimensions. Although the population demonstrates adequate preventive practices, many indifferent attitudes toward dengue prevention persist, which need to be addressed through sustainable educational and sociocultural interventions.

## Resumen

**Introducción:**

El dengue es una enfermedad viral transmitida por la picadura de mosquitos infectados, principalmente Aedes aegypti, que puede ser mortal en casos graves. Estudiar las actitudes y practicas preventivas de la población resulta esencial para fortalecer las estrategias de control y prevención.

**Objetivo:**

Analizar los factores asociados a las actitudes y practicas preventivas del dengue en ciudadanos del norte del Perú.

**Materiales y métodos:**

Se realizó un estudio cuantitativo, no experimental, transversal descriptivo y correlacional. Participaron 295 ciudadanos mayores de 18 años, seleccionados mediante muestreo no probabilístico. Se utilizó un cuestionario validado de 67 ítems, con escala dicotómica para el conocimiento y tipo Likert para las actitudes y prácticas. Se aplicaron análisis descriptivos e inferenciales (V de Cramer y Tau-b de Kendall) a un nivel de confianza del 95%.

**Resultados:**

El 49,5% mostró actitudes indiferentes ante la prevención del dengue, 93,6% presentó prácticas adecuadas y el 54,6% evidenció un alto nivel de conocimiento general sobre la prevención del dengue.

**Discusión:**

La principal actitud más identificada fue el temor a que un familiar contraiga la enfermedad, mientras que las prácticas más frecuentes fueron eliminar criaderos cambiando el agua de los porta-platos y usar repelentes comerciales. Estos hallazgos son similares a los reportados en estudios anteriores.

**Conclusiones:**

Se halló asociación significativa de las actitudes y practicas preventivas, con la mayoría de las dimensiones culturales. Aunque la población demuestra buenas prácticas preventivas, persiste un nivel importante de indiferencia actitudinal que requiere ser abordado mediante intervenciones educativas y socioculturales sostenibles.

## Resumo

**Introdução:**

A dengue é uma doença viral transmitida pela picada de mosquitos infectados, principalmente o Aedes aegypti, que pode ser fatal em casos graves. Estudar as atitudes e práticas preventivas da população é essencial para fortalecer as estratégias de controle e prevenção.

**Objetivo:**

Analisar os fatores associados às atitudes e práticas preventivas em relação à dengue entre cidadãos do norte do Peru.

**Materiais e métodos:**

Foi realizado um estudo quantitativo, não experimental, transversal, descritivo e correlacional. Participaram 295 cidadãos maiores de 18 anos, selecionados por amostragem não probabilística. Foi utilizado um questionário validado de 67 itens, com uma escala dicotômica para conhecimento e uma escala do tipo Likert para atitudes e práticas. Análises descritivas e inferenciais (V de Cramer e Tau-b de Kendall) foram aplicadas com nível de confiança de 95%.

**Resultados:**

49,5% apresentaram atitudes indiferentes em relação à prevenção da dengue, 93,6% demonstraram práticas adequadas e 54,6% apresentaram um alto nível de conhecimento geral sobre a prevenção da dengue.

**Discussão:**

A atitude mais frequentemente identificada foi o medo de que um membro da família contraísse a doença, enquanto as práticas mais comuns foram a eliminação de criadouros por meio da troca da água em escorredores de louça e o uso de repelentes comerciais. Esses achados são semelhantes aos relatados em estudos anteriores.

**Conclusões:**

Foi encontrada uma associação significativa entre atitudes e práticas preventivas e a maioria das dimensões culturais. Embora a população demonstre boas práticas preventivas, persiste um nível significativo de indiferença atitudinal, o que requer intervenção por meio de ações educativas e socioculturais sustentáveis.

## Introduction

Dengue is a disease caused by the bite of infected mosquitoes, primarily Aedes aegypti. This vector is widely distributed across the American continent, particularly in tropical and subtropical regions, with the exception of Canada and continental Chile^1^. Statistics indicate that nearly 50% of the world’s population is at risk of contracting this disease, and between 100 and 400 million infections occur each year. Although many infected individuals are asymptomatic, dengue can be fatal in severe cases^2^.

Symptoms range from mild fever to high debilitating fever, accompanied by severe headache, muscle and joint pain, retro-orbital pain, and erythema. In severe cases, it may cause organ damage and respiratory distress^1^.

The disease occurs primarily during the warm and rainy months. In addition, climate change is facilitating the expansion of vector-borne diseases^3^,^4^. In Peru, the impact of climate change has increased dengue cases, with a case fatality rate of 11%. In response, the government has declared a state of emergency in 11 regions, mainly in the northern part of the country^2^.

Vector control is essential to prevent the spread of the virus, as no specific treatment exists. However, early detection and access to appropriate medical care significantly reduce mortality in complicated cases and severe dengue^1^,^5^. It is necessary to promote communication strategies directed at the population, focusing on preventive measures such as proper washing of water containers, insecticide spraying, use of repellents, and elimination of mosquito breeding sites^5^.

International studies have documented various attitudes, levels of knowledge, and practices related to dengue. In Jazan (Saudi Arabia), demographic factors influenced knowledge and preventive practices, with more favorable attitudes observed among men, although their practices were inadequate^6^. In the Malakand region, healthcare workers showed good knowledge of the vector, mode of dengue transmission, and prevention and control^7^. In Nepal, although residents exhibited a very high level of favorable attitudes, knowledge, and preventive practices were insufficient^8^.

Within this framework, several studies conducted in South American countries have shown how knowledge, attitudes, and practices related to dengue vary according to the sociocultural and geographic context. In Venezuela, high levels of knowledge and favorable attitudes toward dengue were reported. The predominant preventive practices included covering water containers, cleaning the areas around homes, and using insecticides^9^.

In Colombia, nearly 50% of residents had not received training on dengue prevention. However, most individuals were aware of the main symptoms, recognized the severity of the disease, and adopted preventive measures such as cleaning water storage containers and insecticide spraying. Identified risk factors include pet water bowls and water storage tanks^10^. Likewise, sociodemographic variables were not found to be significantly associated with knowledge, attitudes, and practices^11^.

In Brazil, nearly 80% of individuals aged 18 years and older knew what dengue is, and 70% reported taking some preventive measures. However, mosquito breeding sites were identified in nearly 10% of the households of individuals who reported knowing how to prevent the disease^12^.

At the local level, regarding the study of sociodemographic factors associated with knowledge and attitudes toward dengue, Piura, Ucayali, Tumbes, and Lambayeque were the regions of Peru with the highest levels of adequate knowledge of the disease. The regions with the highest levels of preventive attitudes were Madre de Dios, Piura, Cajamarca, and Huancavelica. Most respondents knew the modes of dengue transmission and where to seek help if they developed symptoms. However, a large proportion did not have sufficient knowledge about the symptoms or self-medication. Regarding attitudes, fewer than half of the participants were willing to adopt preventive measures. In addition, being female, being married or living with a partner, and residing in the Peruvian Amazon were associated with better knowledge and preventive attitudes toward dengue. In contrast, being an adolescent or being of Quechua ethnicity was associated with poorer knowledge and unfavorable attitudes toward dengue^13^.

Other studies conducted in Peru indicate that knowledge of dengue is low, regardless of respondents’ age. For example, in an assessment of primary school students with a mean age of 11 years, only 33% had basic knowledge of dengue. This was associated with limited knowledge of the mode of transmission, the characteristics of the vector, the signs and symptoms of the disease, and preventive measures^14^. Similar results were observed in a study conducted among secondary school students in Lambayeque, although in that group, levels of knowledge, attitudes, and practices were higher^15^. When individuals with a mean age of 34 years were assessed, 76% of them exhibited a low level of knowledge, particularly regarding disease transmission, the etiologic agent, symptoms, and warning signs. However, knowledge of preventive measures was intermediate in 93% of the respondents^16^.

Dengue is a public health problem, particularly in northern regions where climatic and social conditions favor the proliferation of the vector. Although the Peruvian Ministry of Health conducts prevention and control campaigns, limitations in knowledge and in the adoption of effective preventive practices among the population persist, contributing to the persistence of mosquito breeding sites and the ongoing transmission of the disease. Accordingly, this study aimed to analyze whether biological, social, and cultural factors are associated with dengue preventive attitudes and practices among citizens in northern Peru. This will help to understand the barriers faced by the community and guide more effective and context-specific interventions to strengthen community participation in reducing the dengue risk.

## Materials and Methods

A quantitative study with a non-experimental, cross-sectional, correlational design was conducted^17^. The study population included citizens aged 18 to 72 years from cities in northern Peru, as this is the area most affected by dengue. The sample consisted of 295 citizens, and the sample size was calculated for a finite population. Considering that an acceptable margin of error commonly used in research ranges between 3% and 6%, a margin of error of 5.6% was used, along with a 95% confidence level. These parameters allowed for the determination of an adequate sample, which was proportionally distributed across the cities in the northern region of the country, as described in the first part of the Results section^18^.

Nonprobability sampling was used. In the first stage, quota sampling was applied, with the sample size distributed proportionally to the population size of each selected department. Subsequently, snowball sampling was used, as each citizen who completed the questionnaire created a multiplier effect by inviting other citizens who met the selection criteria until the required sample size was reached.

The questionnaire developed by the authors was divided into four sections:

First section: It identified the biological and social factors and housing conditions of the citizens, including variables such as age, sex, marital status, educational level, whether they had children, and whether they had stable employment.

Second section: It assessed the cultural dimension through 38 items with response options of Yes, No, and Don’t Know to determine the citizens’ knowledge. This variable included dimensions related to the signs and symptoms of dengue, modes of transmission, knowledge of the disease, and actions to take if symptoms occurred.

Third and fourth sections: They analyzed dengue preventive attitudes and dengue preventive practices through 10 and 12 Likert-scale items, respectively. Preventive practices were evaluated in two dimensions: prevention of mosquito bites and prevention of mosquito breeding sites. In addition, sources of information about dengue were identified, as well as whether participants had been infected and the type of dengue they had contracted.

To categorize the levels of knowledge, attitudes, and practices related to dengue prevention, a scoring procedure was used to establish cutoff points to classify the results into low, moderate, and high levels (knowledge); unfavorable, indifferent, and favorable (attitudes); and adequate or inadequate (practices).

The scoring procedure involved identifying the minimum and maximum scores obtained on each scale. Based on these values, the total range was calculated as the difference between the minimum and maximum scores, which was then divided by the number of categories established for each variable, yielding the corresponding intervals for each level.

Based on these intervals, participants’ scores were classified according to the established score ranges, allowing for an objective, consistent, and reproducible interpretation of the results. This process enabled the clear identification of the different levels of knowledge as well as the attitudes and practices related to dengue prevention.

The data collection instrument was validated through expert judgment by five health professionals (one epidemiologist, one nurse, two microbiologists, and one physician), who provided comments to improve the instrument. Aiken’s V was 0.94, indicating acceptable validity. Reliability analysis was conducted using questionnaires from a pilot sample of 45 participants from the different departments included in the study. The results showed acceptable reliability: the items for the knowledge variable had a Cronbach’s alpha of 0.90. The attitude and practice items were analyzed using McDonald’s omega coefficient^19^, with coefficients of 0.78 for attitudes and 0.86 for preventive practices. For data collection, the questionnaire was designed in Google Forms® and administered online to the required sample between January and March 2024.

The quality of the data collected through the online questionnaire was verified prior to processing and analysis.

**Statistical analysis**

The database was exported to SPSS, version 27. To identify the biological, social, housing, and cultural factors associated with attitudes and practices related to dengue prevention, statistical tests of association were performed according to the nature of the variables. For nominal categorical variables, Cramér’s V coefficient was used; for ordinal categorical variables, Kendall’s Tau-b coefficient was applied, with Monte Carlo simulation at a 95% confidence level^20^. The complete dataset collected in this study is available for open access in the Zenodo repository^21^.

**Ethical considerations**

The study was approved by the Ethics Committee of the Professional School of Medicine under (Approval No. 363-CEI-EPM-UCV-2023). In addition, the principles of the Declaration of Helsinki (respect, beneficence, and justice) were observed. Informed consent was prioritized and included in the Google Form prior to the start of the questionnaire, and participants’ anonymity and integrity were ensured^22^.

## Results

In each stratum, respondents were selected proportionally according to the estimated population provided by the National Institute of Statistics and Informatics for northern Peru. The sample distribution was as follows: 15 respondents from Tumbes (5.08%), 40 from Piura (13.56%), 57 from Lambayeque (19.32%), 53 from La Libertad (17.97%), 34 from Ancash (11.53%), 50 from Cajamarca (16.95%), 37 from San Martín (12.54%), and 9 from Amazonas (3.05%)^18^.

Participant characteristics included a mean age of 34.69 years with a standard deviation of 14.46 years. A total of 51.86% of participants were younger than 30 years, 58.98% were female, 59.66% were single, 92.20% had higher education, 44.75% did not have children, and 58.31% did not have stable employment Table 1.

**Figure 1:**
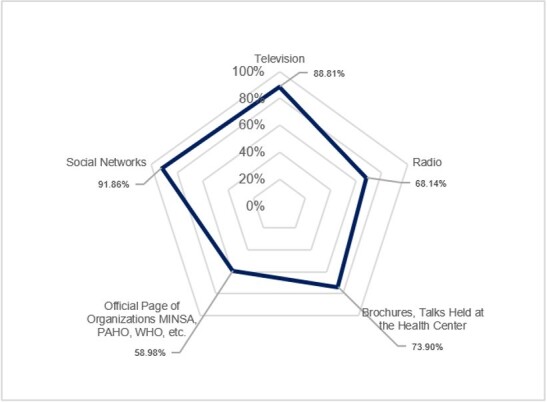
Sources of information through which participants learn about dengue

As shown in Figure 1, most participants reported obtaining information from social media (91.86%), followed by television (88.81%). Additionally, 73.90% indicated that they obtained information through brochures and talks at health centers, among other sources.

Table 1 shows that 49.15% of participants had a good preventive attitude toward dengue. Among those with a good attitude, 30.51% were female, 29.15% were single, 45.08% had higher education, 23.05% had children, and 28.14% did not have stable employment. On the other hand, no statistically significant association was found between biological and social factors and attitudes toward dengue prevention (p> 0.05). Likewise, among participant with a good attitude, 43.73% reported having daily access to drinking water, 44.07% had sewer or drainage services, 37.97% had a cistern or elevated water tank, 15.93% stored water in buckets or containers, 19.66% had plants or flowers kept in water, 7.12% had an aquarium, pool, or well, 35.93% reported that their homes had roofs made of solid materials, 7.12% reported having items such as tires or pipes on the roof, 30.17% reported having daily garbage collection service, and 10.51% lived in flood-prone areas. Similarly, none of the housing characteristics were found to be significantly associated with dengue preventive attitudes among citizens in northern Peru (p> 0.05).

Table 1 also shows that 30.51% of the surveyed citizens in northern Peru had a high level of overall knowledge of dengue and a good preventive attitude. Among the cultural factors, almost all dimensions showed statistically significant associations (p< 0.05), except for the level of knowledge regarding modes of transmission, which did not show a statistically significant association with attitudes toward dengue prevention (p> 0.05).

**Table 1:** Factors associated with dengue preventive attitudes among citizens in northern Peru

Factor	Variable / Category	Attitude			Total % (n=295)	Sig.
		Unfavorable % (n = 4)	Indifferent % (n=146)	Favorable % (n=145)		
Biological	Sex					0.252^a^
	Male	1.02 (3)	21.36 (63)	18.64 (55)	41.02 (121)	
	Female	0.34 (1)	28.14 (83)	30.51 (90)	58.98 (174)
	Age					0.796^b^
	Under 30 years	0.34 (1)	26.10 (77)	25.42 (75)	51.86 (153)	
	30-59 years	1.02 (3)	19.66 (58)	21.36 (63)	42.03 (124)
	60 years or older	0.00 (0)	3.73 (11)	2.37 (7)	6.10 (18)	
	Marital status					0.631^a^
	Single	0.34 (1)	30.17 (89)	29.15 (86)	59.66 (176)	
	Married or cohabiting	1.02 (3)	16.61 (49)	17.29 (51)	34.92 (103)	
	Divorced or separated	0.00 (0)	1.69 (5)	2.37 (7)	4.07 (12)
	Widowed	0.00 (0)	1.02 (3)	0.34 (1)	1.36 (4)	
	Has children					0.321^a^
	Yes	1.02 (3)	20.68 (61)	23.05 (68)	44.75 (132)	
	No	0.34 (1)	28.81 (85)	26.10 (77)	55.25 (163)	
	Educational level					0.711^b^
	Primary or secondary	0.00 (0)	3.73 (11)	4.07 (12)	7.80 (23)	
	Higher education	1.36 (4)	45.76 (135)	45.08 (133)	92.20 (272)	
	Has stable employment					0.346^a^
	Yes	1.02 (3)	19.66 (58)	21.02 (62)	41.69 (123)	
	No	0.34 (1)	29.83 (88)	28.14 (83)	58.31 (172)	
Housing conditions	Daily access to drinking water (Yes)	1.36 (4)	43.05 (127)	43.73 (129)	88.14 (260)	0.664^a^
	Sewer or drainage service available (Yes)	1.36 (4)	45.42 (134)	44.07 (130)	90.85 (268)	0.669^a^
	Water tank or cistern available (Yes)	1.36 (4)	36.61 (108)	37.97 (112)	75.93 (224)	0.425^a^
	Stores water in buckets or containers (Yes)	0.68 (2)	17.29 (51)	15.93 (47)	33.90 (100)	0.714^a^
	Plants or flowers kept in water (Yes)	0.68 (2)	21.02 (62)	19.66 (58)	41.36 (122)	0.858^a^
	Aquarium, pool, or well present (Yes)	0.34 (1)	6.44 (19)	7.12 (21)	13.90 (41)	0.760^a^
	Roof made of solid material (Yes)	1.02 (3)	37.63 (111)	35.93 (106)	74.58 (220)	0.849^a^
	Items such as tires or pipes on the roof (Yes)	0.34 (1)	3.73 (11)	7.12 (21)	11.19 (33)	0.116^a^
	Garbage collection occurs daily (Yes)	1.02 (3)	29.15 (86)	30.17 (89)	60.34 (178)	0.759^a^
	Lives in flood-prone areas (Yes)	0.34 (1)	10.51 (31)	10.51 (31)	21.36 (63)	0.984^a^
Cultural factors	Overall knowledge level					0.007^b^
	Low	0.34 (1)	3.39 (10)	1.02 (3)	4.75 (14)	
	Moderate	0.00 (0)	23.05 (68)	17.63 (52)	40.68 (120)
	High	1.02 (3)	23.05 (68)	30.51 (90)	54.58 (161)	
	Knowledge of symptoms					0.031^b^
	Low	0.34 (1)	10.17 (30)	5.76 (17)	16.27 (48)	
	Moderate	0.34 (1)	15.25 (45)	14.24 (42)	29.83 (88)	
	High	0.68 (2)	24.07 (71)	29.15 (86)	53.90 (159)	
	Knowledge of transmission modes					0.981^b^
	Low	0.34 (1)	2.71 (8)	2.71 (8)	5.76 (17)	
	Moderate	0.00 (0)	22.71 (67)	22.03 (65)	44.75 (132)	
	High	1.02 (3)	24.07 (71)	24.41 (72)	49.49 (146)	
	Knowledge of the disease					0.001^b^
	Low	0.34 (1)	9.49 (28)	4.41 (13)	14.24 (42)	
	Moderate	0.68 (2)	26.44 (78)	24.07 (71)	51.19 (151)	
	High	0.34 (1)	13.56 (40)	20.68 (61)	34.58 (102)	
	Knowledge of actions to take					0.046^b^
	Low	0.00 (0)	1.36 (4)	0.34 (1)	1.69 (5)	
	Moderate	0.68 (2)	27.46 (81)	22.71 (67)	50.85 (150)	
	High	0.68 (2)	20.68 (61)	26.10 (77)	47.46 (140)	
	Total	1.36 (4)	49.49 (146)	49.15 (145)	100 (295)	

a: Cramér’s V test with Monte Carlo simulation at 95% confidence level. b: Kendall’s Tau-b test with Monte Carlo simulation at 95% confidence level.

Table 2 shows that 93.56% of participants demonstrated adequate preventive practices, while only 6.44% exhibited inadequate preventive practices. Additionally, none of the biological or social factors were statistically associated with dengue preventive practices (p> 0.05).

Among respondents with adequate dengue preventive practices, 82.71% had daily access to drinking water, 85.42% had sewer or drainage services, 71.19% had a cistern or elevated water tank, and 31.86% stored water in buckets or containers. Additionally, 1.36% had inadequate practices and reported having plants or flowers kept in water. Finally, no housing characteristics were found to be significantly associated with dengue preventive practices (p> 0.05). These results are presented in Table 2.

Regarding the cultural factors related to dengue preventive practices, no statistically significant associations were identified (p> 0.05), as shown in Table 2.

**Table 2:** Factors associated with dengue preventive practices among citizens in northern Peru

Factor	Variable / Category	Dengue preventive practices		Total % (n = 295)	Sig.
		Inadequate % (n = 19)	Adequate % (n = 276)		
Biological and social factors	Sex				0.561^a^
	Male	3.05 (9)	37.97 (112)	41.02 (121)	
	Female	3.39 (10)	55.59 (164)	58.98 (174)	
	Age				0.189^a^
	Under 30 years	3.05 (9)	48.81 (144)	51.86 (153)	
	30-59 years	2.37 (7)	39.66 (117)	42.03 (124)	
	60 years or older	1.02 (3)	5.08 (15)	6.10 (18)	
	Marital status				0.467^a^
	Single	3.39 (10)	56.27 (166)	59.66 (176)	
	Married or cohabiting	2.37 (7)	32.54 (96)	34.92 (103)	
	Divorced or separated	0.68 (2)	3.39 (10)	4.07 (12)	
	Widowed	0.00 (0)	1.36 (4)	1.36 (4)	
	Has children				0.475^a^
	Yes	3.39 (10)	41.36 (122)	44.75 (132)	
	No	3.05 (9)	52.2 (154)	55.25 (163)	
	Educational level				0.670^a^
	Primary or secondary	0.34 (1)	7.46 (22)	7.80 (23)	
	Higher education	6.10 (18)	86.10 (254)	92.20 (272)	
	Has stable employment				0.970^a^
	Yes	2.71 (8)	38.98 (115)	41.69 (123)	
	No	3.73 (11)	54.58 (161)	58.31 (172)	
Housing conditions	Daily access to drinking water	5.42 (16)	82.71 (244)	88.14 (260)	0.584^a^
	Sewer or drainage service available	5.42 (16)	85.42 (252)	90.85 (268)	0.300^a^
	Cistern or elevated water tank available	4.75 (14)	71.19 (210)	75.93 (224)	0.813^a^
	Stores water in buckets or containers	2.03 (6)	31.86 (94)	33.9 (100)	0.825^a^
	Plants or flowers kept in water	1.36 (4)	40.00 (118)	41.36 (122)	0.063^a^
	Aquarium, pool, or well present	0.00 (0)	13.90 (41)	13.90 (41)	0.070^a^
	Roof made of solid construction material	4.75 (14)	69.83 (206)	74.58 (220)	0.926^a^
	Items such as tires or pipes on the roof	0.34 (1)	10.85 (32)	11.19 (33)	0.397^a^
	Daily garbage collection service	4.07 (12)	56.27 (166)	60.34 (178)	0.795^a^
	Lives in flood-prone areas	1.02 (3)	20.34 (60)	21.36 (63)	0.540^a^
Cultural Factors	Overall knowledge level				0.521^a^
	Low	0.34 (1)	4.41 (13)	4.75 (14)	
	Moderate	3.39 (10)	37.29 (110)	40.68 (120)	
	High	2.71 (8)	51.86 (153)	54.58 (161)	
	Knowledge of symptoms				0.203^a^
	Low	1.02 (3)	15.25 (45)	16.27 (48)	
	Moderate	3.05 (9)	26.78 (79)	29.83 (88)	
	High	2.37 (7)	51.53 (152)	53.90 (159)	
	Knowledge of transmission modes				0.590^a^
	Low	0.68 (2)	5.08 (15)	5.76 (17)	
	Moderate	3.05 (9)	41.69 (123)	44.75 (132)	
	High	2.71 (8)	46.78 (138)	49.49 (146)	
	Knowledge of the disease				0.384^a^
	Low	1.36 (4)	12.88 (38)	14.24 (42)	
	Moderate	3.73 (11)	47.46 (140)	51.19 (151)	
	High	1.36 (4)	33.22 (98)	34.58 (102)	
	Knowledge of actions to take				0.378^a^
	Low	0.34 (1)	1.36 (4)	1.69 (5)	
	Moderate	2.71 (8)	48.14 (142)	50.85 (150)	
	High	3.39 (10)	44.07 (130)	47.46 (140)	
	Total	6.44 (19)	93.56 (276)	100 (295)	

a: Cramer's V test with Monte Carlo simulation at 95% confidence level.

**Figure 2:**
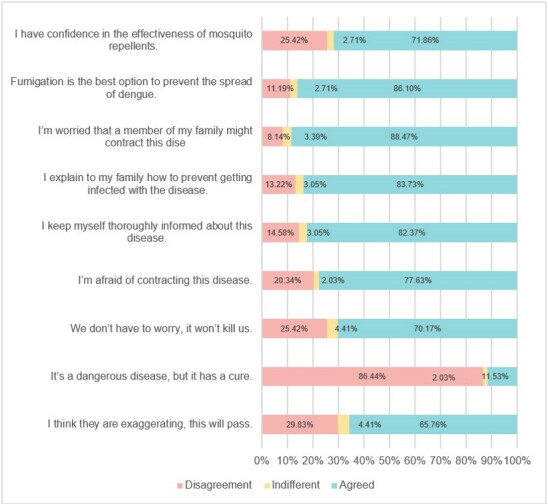
Attitudes toward dengue prevention

Figure 2 shows that the most frequently reported attitude was concern that a household member could contract the disease, with 88.47% agreeing. In contrast, the statement that dengue is a dangerous disease but has a cure showed the highest level of disagreement.

**Figure 3:**
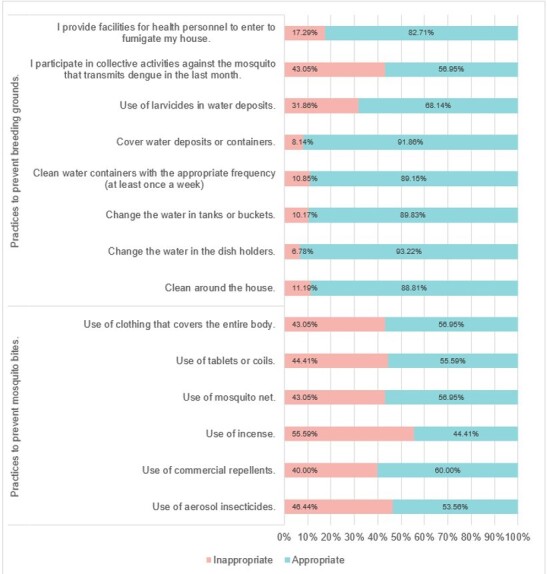
Practices for dengue prevention

Figure 3 presents the practices used to prevent dengue. The most frequent adequate practice to prevent mosquito breeding sites was changing the water in plant saucers (93.22%), while the most frequent practice to prevent mosquito bites was the use of commercial insect repellents (60%).

## Discussion

The results presented in Figure 1 revealed that social media and television are important sources of information about dengue. These findings are consistent with those reported in the Philippines, where television was identified as the most reliable source of information^23^. Similarly, in Malaysia, social media were found to be among the most relevant sources of information about dengue, particularly among younger populations^24^. In addition, another study reported that mass media, such as television and radio, were the main sources of information about dengue^25^,^26^. However, in Thailand, a large proportion of participants indicated that their primary source of information was teachers, followed by parents and television^27^.

In this context, the results highlight the importance of social media and current mass media. Therefore, these channels should be considered when designing communication and educational strategies, taking into account the economic, social, and cultural characteristics of the population. An empowered population can become a new “vector” of information and a multiplier of this knowledge^28^.

Women had better preventive attitudes toward dengue, as reported in Malaysia, where women showed more favorable attitudes than men^29^,^30^. This finding differs from what was observed in Saudi Arabia, where men demonstrated more favorable attitudes^5^. In Peru, women were also found to have more favorable preventive attitudes^13^. Although no statistically significant association was identified between these variables, relevant percentages were observed that could suggest a possible association. Therefore, it is crucial that health policies take these factors into account, as they directly or indirectly influence behavior regarding this disease^31^.

Regarding housing characteristics, these do not appear to determine dengue preventive attitudes among citizens in northern Peru. This result differs from a study conducted in Ecuador, which concluded that the most appropriate model for predicting the presence of Aedes aegypti included variables related to housing conditions^32^. This is also associated with socioeconomic factors, as socially vulnerable individuals have a greater likelihood of experiencing some stage of the disease^33^,^34^.

Attitudes toward dengue prevention in northern Peru were categorized as indifferent or favorable, in contrast to countries such as Nepal, Malaysia, and Brazil, where participants showed more favorable attitudes toward prevention^8^,^12^,^35^. In this study, attitudes were found to be significantly associated with educational level and employment status, whereas preventive practices were related to variables such as age, marital status, geographic location, and level of knowledge^29^. Additionally, previous experience with dengue was positively associated with more favorable attitudes toward its prevention, while poor knowledge and unfavorable attitudes were linked to lower educational levels^30^.

Overall knowledge levels, as well as knowledge related to symptoms and modes of transmission, were predominantly high, similar to what has been observed in the Philippines, where respondents demonstrated a good understanding of dengue transmission modes, signs and symptoms, prevention, and control^22^. In contrast, in Malaysia, most participants had a moderate level of knowledge^34^. In Sri Lanka, good knowledge was found to be significantly associated with individuals aged between 41 and 60 years, secondary or higher education, and higher monthly income^30^.

Among the frequently reported attitudes were concern that a family member could contract the disease, acceptance of insecticide spraying as a preventive measure against dengue, and willingness to explain to family members how to prevent infection. Regarding preventive practices, most respondents reported adequate preventive behaviors. Among the practices used to prevent mosquito breeding sites, the most notable were changing the water in plant saucers, covering water storage containers, and regularly changing the water in tanks or buckets. Regarding the prevention of mosquito bites, the most frequently reported strategies included the use of commercial insect repellents and mosquito nets, and wearing clothing that covers the entire body. These findings are similar to those reported in studies conducted in Venezuela, Colombia, and Ecuador, where preventive measures included covering water storage containers, cleaning the surroundings of homes, and using insecticides^9^,^10^,^32^. In Sri Lanka, most participants did not have mosquito breeding sites, while a smaller proportion reported the presence of larvae and eggs in trays and flower pots. Most participants also kept their garbage containers closed, regularly cleaned water containers, and used larvicides^30^.

These findings may inform the development of social mobilization and dengue control strategies by addressing gaps and barriers related to knowledge, preventive attitudes, and preventive practices in vulnerable areas^36^. It is essential to focus on proactive communication campaigns and training programs that strengthen preventive measures, such as insecticide spraying and other effective practices.

The main limitation of this study was the data collection process, due to the low participation among citizens in completing the questionnaire. The strategy used, which was based on nonprobability sampling, limits the generalizability of the results. However, the adequate sample size, together with an acceptable margin of error and the appropriate use of statistical techniques, allows for reliable estimates that provide an approximation of the behavior of the variables in the population studied. One of the main strengths of the study is the design and development of the questionnaire by the authors, which allowed the items to be tailored to the characteristics of the study population. The study also adopts a comprehensive approach by considering biological, social, and cultural factors in understanding dengue preventive attitudes and practices. In addition, the regional contextualization provides useful evidence to guide public health interventions adapted to the context of northern Peru.

## Conclusions

Biological and social factors, as well as housing conditions, were not significantly associated with dengue preventive attitudes or practices among citizens in northern Peru. This suggests that these factors do not determine preventive attitudes or practices related to dengue.

Most of the cultural dimensions analyzed showed a statistically significant association with preventive attitudes and practices, except for the level of knowledge regarding modes of transmission. This finding indicates that, although individuals may have greater or lesser knowledge about how the disease is transmitted, this does not necessarily change their attitudes or preventive practices.

The most prominent attitude was concern that a family member could contract the disease, indicating a high level of sensitivity to risk and the importance placed on family well-being as a central element in the response to the disease.

Among preventive practices, the most common measure to prevent mosquito breeding sites was changing the water in plant saucers, while the use of commercial insect repellents stood out as the most widely adopted strategy to prevent mosquito bites. These preventive actions are consistent with what has been reported in the literature.
